# Mechanical Debridement with Antibiotics in the Treatment of Chronic Periodontitis: Effect on Systemic Biomarkers―A Systematic Review

**DOI:** 10.3390/ijerph17155601

**Published:** 2020-08-03

**Authors:** Sudhir L. Munasur, Eunice B. Turawa, Usuf M.E. Chikte, Alfred Musekiwa

**Affiliations:** 1 Division of Epidemiology and Biostatistics, Department of Global Health, Faculty of Medicine and Health Sciences, Stellenbosch University, Cape Town 7530, South Africa; munasurs@gmail.com (S.L.M.); Eunice.Turawa@mrc.ac.za (E.B.T.); 2 Division of Health Systems and Public Health, Department of Global Health, Faculty of Medicine and Health Sciences, Stellenbosch University, Cape Town 7530, South Africa; umec@sun.ac.za

**Keywords:** chronic periodontitis, mechanical debridement, antibiotics, systemic biomarkers

## Abstract

In this systematic review, we assessed the effectiveness of systemic antibiotics as an adjunctive therapy to mechanical debridement in improving inflammatory systemic biomarkers, as compared to mechanical debridement alone, among adults with chronic periodontitis. We searched relevant electronic databases for eligible randomized controlled trials. Two review authors independently screened, extracted data, and assessed risk of bias. We conducted meta-analysis, assessed heterogeneity, and assessed certainty of evidence using GRADEPro software. We included 19 studies (n = 1350 participants), representing 18 randomized controlled trials and found very little or no impact of antibiotics on inflammatory biomarkers. A meta-analysis of eight studies demonstrated a mean reduction of 0.26 mm in the periodontal pockets at three months (mean difference [MD] −0.26, 95%CI: −0.36 to −0.17, n = 372 participants, moderate certainty of evidence) in favor of the antibiotics. However, results from five studies reporting clinical attachment level (mm) yielded little or no difference at three months (MD −0.16, 95% CI: −0.35 to 0.03, n = 217 participants) between antibiotic and placebo groups. There is little or no evidence that adjunctive systemic antibiotics therapy improves inflammatory systemic biomarkers, compared to mechanical debridement alone, among adults with chronic periodontitis.

## 1. Background

According to the World Health Organization (WHO), oral diseases are the most common non-communicable diseases with severe periodontal disease being the 11th most prevalent disease worldwide [1]. Periodontal disease is a chronic inflammatory disease of the periodontium and the most prevalent infectious oral condition [2]. Although the disease can be treated and prevented, it is the most frequent cause of tooth loss in adults. The signs and symptoms of periodontitis include persistent halitosis, red or swollen gingiva, tender or bleeding gingiva, painful mastication, loose teeth, and gingival recession. A new identification and classification system of periodontitis was developed in 2017 whereby the 1999 classification of chronic periodontitis is incorporated under the category of periodontitis (previously considered as either chronic or aggressive) [3]. The prevalence of periodontitis ranges 5–15% in most populations [4]. Globally, periodontal disease had an estimated prevalence of 750,847,000, incidence of 89,840,000, and 4,898,000 years lived with disability (YLD), in 2016 [5]. 

The diagnosis of periodontal disease is based on clinical measures such as bleeding on probing, pocket probing depth, and attachment loss, as well as radiological evidence of bone destruction [6]. Periodontal treatment entails the elimination of biofilm and microbial deposits from the root surfaces, thus reducing the inflammatory host response and tissue destruction [7,8]. Although there are several treatment approaches for periodontitis, conservative mechanical debridement (scaling and root planing [SRP]) has been the most common therapy [9]. Depending on the severity of inflammation, mechanical debridement combined with systemic antibiotic use has been advocated as a treatment possibility. However, there is currently insufficient scientific evidence to support or refute whether systemic antibiotics effectively enhances a positive outcome in chronic periodontal disease [10,11,12,13,14,15]. 

Therefore, this systematic review assessed the effectiveness of systemic antibiotics as an adjunctive therapy to mechanical debridement in the improvement of inflammatory systemic biomarkers, as compared to mechanical debridement alone, among adults with chronic periodontitis, using evidence from published randomized controlled trials.

## 2. Methods

This systematic review followed the preferred reporting items for systematic reviews and meta-analyses (PRISMA) statement for reporting systematic reviews and meta-analyses of studies that evaluate healthcare interventions [16]. The protocol for study was registered with the International Prospective Register of Systematic Reviews (PROSPERO), (registration number: RD42017059053). 

### 2.1. Criteria for Considering Studies for This Review

#### 2.1.1. Types of Studies

We included all randomized controlled trials (RCTs), published between January 2000 and October 2019, evaluating systemic antibiotics combined with mechanical debridement versus mechanical debridement alone or with placebo. Quasi-randomized controlled trials were also eligible, as well as studies in abstract only, with relevant results. Non-randomized trials and observational studies met the exclusion criteria.

#### 2.1.2. Types of Participants

We included adults, aged 18 years and older, diagnosed with periodontal disease with or without co-morbidities such as diabetes mellitus, cardiovascular diseases, chronic obstructive pulmonary disease, and chronic kidney disease.

#### 2.1.3. Types of Interventions

##### Experimental

All interventions that included mechanical debridement combined with adjunctive systemic antibiotics for the treatment of chronic periodontitis.

##### Control

The trials compared antibiotics versus placebo or no antibiotic, with mechanical debridement in either arm of the intervention.

#### 2.1.4. Types of Outcome Measures

##### Primary Outcomes

The primary outcomes were changes in blood levels of inflammatory biomarkers such as Matrix Metalloproteinases (MMPs), Tissue Inhibitors of MMPs (TIMPs), Cytokines/Interleukins (IL-1β, IL-6 and IL-8), C-Reactive Protein (CRP), Glycosylated Hemoglobin (HbA1c), and Tumor Necrosis Factor alpha (TNF-α).

##### Secondary Outcomes

The secondary outcomes were periodontal parameters including pocket depth and clinical attachment level (CAL).

### 2.2. Search Methods for Identification of Studies

We adopted the following search terms and strategy: "anti-bacterial agents” [medical subject headings (MeSH)] OR "anti-infective" OR “systemic antibiotics” OR “antibiotic OR "antibiotic therapy”) AND (periodontitis OR “chronic periodontitis” OR “periodontal diseases” [MeSH] OR “periodontitis” [MeSH]. 

We used the search strategy to identify relevant trials in MEDLINE database, and adapted the same strategy for other relevant electronic databases that we searched. We considered relevant RCTs published from January 2000 to October 2019 for inclusion. We did not apply any language restrictions.

#### 2.2.1. Electronic Searches

We searched the following databases to identify relevant trials: Cochrane Oral Health Group’s Trials Register, CENTRAL—Cochrane Register of Controlled Trials (of the Cochrane Library—current issue), MEDLINE (1966 to present), EMBASE (1982 to present), CINAHL (1990 -present), and Google scholar (1990–present).

#### 2.2.2. Ongoing Trials Databases

We searched the following on-going trials registers (31 October 2019) to identify relevant trials: The meta-Register of Controlled Trials (www.controlled-trials.com), the US National Institutes of Health On-going Trials Register (www.clinicaltrials.gov), the World Health Organization International Clinical Trials Registry platform (www.who.int/trialsearch). 

#### 2.2.3. Grey Literature

We also searched the reference lists of included trials (31 October 2019) for relevant trials. We also emailed the authors of included trials and experts in the field of oral health care to identify any additional published or unpublished trials (31 October 2019). We searched the ProQuest database, Stellenbosch University database, and Google scholar (31 October 2019) and hand searched for trials not indexed in databases. 

### 2.3. Data Collection and Analysis

#### 2.3.1. Selection of Studies

Two review authors (SM and ET) independently screened the titles and abstracts of the search output to select potentially eligible trials using pre-specified eligibility criteria. After removing duplicates and ineligible trials, we retrieved the full-text articles of potentially relevant trials. Any disagreements were resolved through discussion or the third and fourth review authors (UMEC and AM) would add their input to enable a consensus. 

#### 2.3.2. Data Extraction and Management

Two review authors (SM and ET) also independently extracted data from each included trial using a pre-piloted data extraction form specifically designed for this review. The extracted data included study characteristics (authors, year, country and setting, and funding) and participant characteristics (study population, age, gender, periodontal disease diagnosis and severity, number of participants recruited and number of participants completing the trial, withdrawals and the reasons thereof, and overall sample size). Three review authors (SM, ET, and AM) extracted data for primary and secondary outcomes. For continuous outcomes that could be assumed normally distributed, such as pocket depth (in mm), we extracted the mean and standard deviation. For other continuous outcomes containing outliers, such as IL-1β, IL-6, IL-8, CRP, and CAL, we extracted the medians and their corresponding inter-quartile ranges (IQR). Otherwise, we extracted any other relevant data such as median ratios, 95% confidence intervals (CI), and p-values, as reported by study authors.

#### 2.3.3. Assessment of Risk of Bias of Included Studies

Two review authors (SM and ET) independently assessed the risk of bias assessment for each of the included studies, in accordance with the guidelines in the Cochrane Handbook for Systematic Reviews of Interventions and levels of attrition were noted [17]. Each domain of risk of bias (generation of allocation sequence, allocation concealment, blinding of participants and personnel, blinding of outcome assessors, incomplete outcome data, selective outcome reporting, and other bias) were judged as either low risk, high risk, or unclear. Disagreements were resolved by discussion or by consulting AM.

#### 2.3.4. Measures of Treatment Effect

We calculated the mean difference (MD), with its corresponding 95%CI, for continuous data with the same scale (for instance, pocket depth (mm)). Where continuous data could not be assumed to be normally distributed and study authors reported medians and ranges (for instance, CRP), we reported the median (IQR) together with any reported p-values for group comparisons, as given by study authors. For binary data, the reported odds ratios (OR) and corresponding 95% CI’s and p-values were reported. 

#### 2.3.5. Unit of Analysis Issues

We performed analyses at the participant level to avoid unit of analysis errors. We did not include any crossover or cluster-randomized trials. For trials that had multiple intervention groups, we combined relevant groups to create single pairwise comparison. For one three-arm trial, we selected one pair of interventions and excluded one arm that was not relevant for this review.

#### 2.3.6. Dealing with Missing Data

We contacted study authors to request information regarding missing data on either outcomes or risk of bias; however, we did not receive any responses. For each outcome in each trial, the denominator was the number of randomized subjects excluding any participants whose outcomes were missing. We therefore used the available case analysis and did not perform any data imputation. 

#### 2.3.7. Data Synthesis

We performed inverse variance random effects meta-analysis using RevMan statistical software version 5.3 (The Nordic Cochrane Centre, Copenhagen, Denmark) for some continuous secondary outcomes such as pocket depth (mm). To assess the extent and significance of heterogeneity, we used the I^2^ test statistic and the Chi^2^ test (*p* < 0.1 indicated statistical significance). Due to insufficient data, we could not pool results of many outcomes in a meta-analysis, and we reported the results separately for each study. We used GRADE Profiler (GRADEpro, version 3.6) software to assess the quality of evidence on the four main outcomes (HbA1c, MMP-8, CRP, and probing depth at 3 months) by rating the quality of evidence as either high, moderate, low, and very low certainty of evidence and summarized the results in the Summary of Findings table. Grading of the evidence considers factors such as study limitation (risk of bias), imprecision, inconsistency, indirectness of results, and publication bias [18].

## 3. Results

### 3.1. Results of the Search

**Figure 1:**
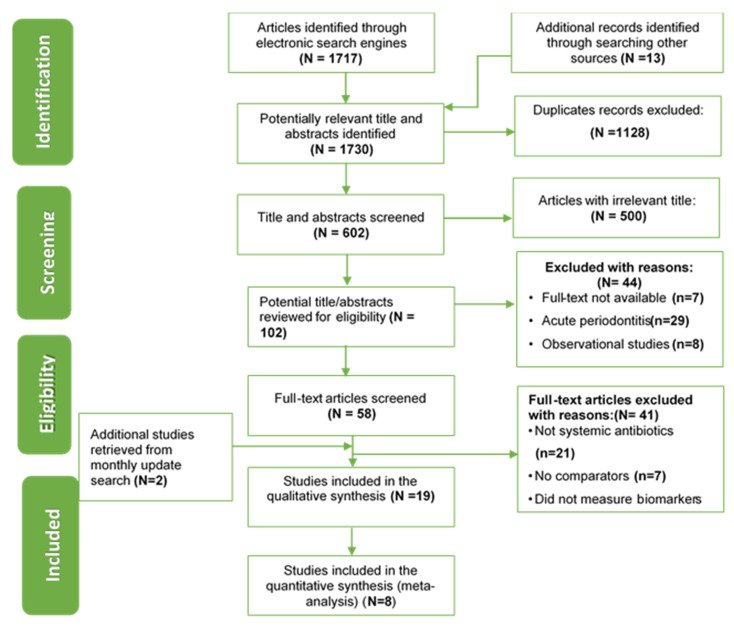
Search results for studies on the effect of antibiotics in addition to mechanical debridement among adults with chronic periodontitis

The search yielded a total of 1730 titles and abstracts (1717 from electronic searches and 13 from hand searches). We excluded 1128 duplicates and remained with 602 records. We screened these 602 titles and abstracts and retained 58 records for further assessment. We also identified two additional articles from an updated search. We then retrieved full-text articles of these 60 studies and re-screened them for eligibility, from which we excluded 41 studies with reasons, thus retaining with 19 studies that we included in this review [13,15,19,20,21,22,23,24,25,26,27,28,29,30,31,32,33,34,35]. We report on 18 randomized controlled trials considering that two studies [26,31] were from the same trial. One trial [35] was published in French and we obtained an English translation. (Figure 1). 

### 3.2. Settings, Participants, and Interventions

The characteristics of included studies are summarized in Table 1.

The trials were conducted in academic hospitals mainly in developed countries: Six from the USA [22,25,26,28,31,32]; five from South America (Colombia [20,35], Brazil [15,30] and Chile [29]); five from Europe (Switzerland [19,21], Poland [24], Turkey [13,27]); and one from Australia [33]. Only one study was from a developing country, India [23]. None of the included studies were conducted in Africa.

In total, 1350 participants (18 trials) were included in the analysis for this review. The age of the participants ranged from 18 to 75 years old. Participants were all diagnosed with moderate-to-advanced chronic periodontitis with or without comorbidities. Eight trials included participants with no comorbidities [19,21,25,27,32,33,34,35]. Seven trials [15,20,22,23,24,28,30] included participants with hyperglycemia (diabetes), one trial [13] recruited participants with coronary artery disease (CAD), another trial [29] included participants with metabolic syndrome (MetS), and the last trial reported by two studies [26,31] assessed osteopenic postmenopausal women (Table 1).

All included trials assessed the effect of antibiotics compared with placebo or no antibiotic with mechanical debridement in both groups. The duration of the trials ranged from 6 weeks to 18 months. The intervention groups had broad spectrum antibiotics as an adjunct to the non-surgical therapies received. Combination of interventions varied across included trials. Four trials [20,27,33,35] evaluated the effect of 500 mg azithromycin in the treatment group compared to placebo. Seven trials examined the effects of doxycycline against either a placebo [15,22,25,26] or no placebo [23,24,28]. Seven trials investigated the effect of a combination of metronidazole and amoxicillin, albeit utilizing different dosages, versus either placebo [21,29,30,32,33,34] or no placebo [19] (Table 1). 

Some of the trials were multi-arm. One trial [20] randomized patients into three treatment groups: Non-surgical therapy plus azithromycin, non-surgical therapy plus placebo, and supragingival prophylaxis plus azithromycin; we analyzed the first two groups and excluded the third group since it had no SRP. The second trial [22] was also a three-arm trial with conventional subgingival debridement combined with either sub-antimicrobial-dose doxycycline (SDD), anti-microbial dose doxycycline (ADD), or placebo. The third trial [25] consisted of four groups of various 20 mg doxycycline prescriptions versus placebo. The fourth trial [32] was a four-arm trial where all patients received quadrant SRP and were then prescribed 250 mg amoxicillin and 200 mg metronidazole (AP) or lactate and metronidazole (PM) or amoxicillin and calcium lactate (AP) or lactate and calcium lactate (PP). The fifth trial [33] is a three-arm trial, the first group received 200 mg metronidazole plus 500 mg amoxicillin, the second group were given 500 mg azithromycin, and the control group received placebo. For the analysis of these multi-arm trials, we combined the antibiotic groups to create single pairwise comparison with the placebo.

### 3.3. Risk of Bias of Included Studies

**Figure 2:**
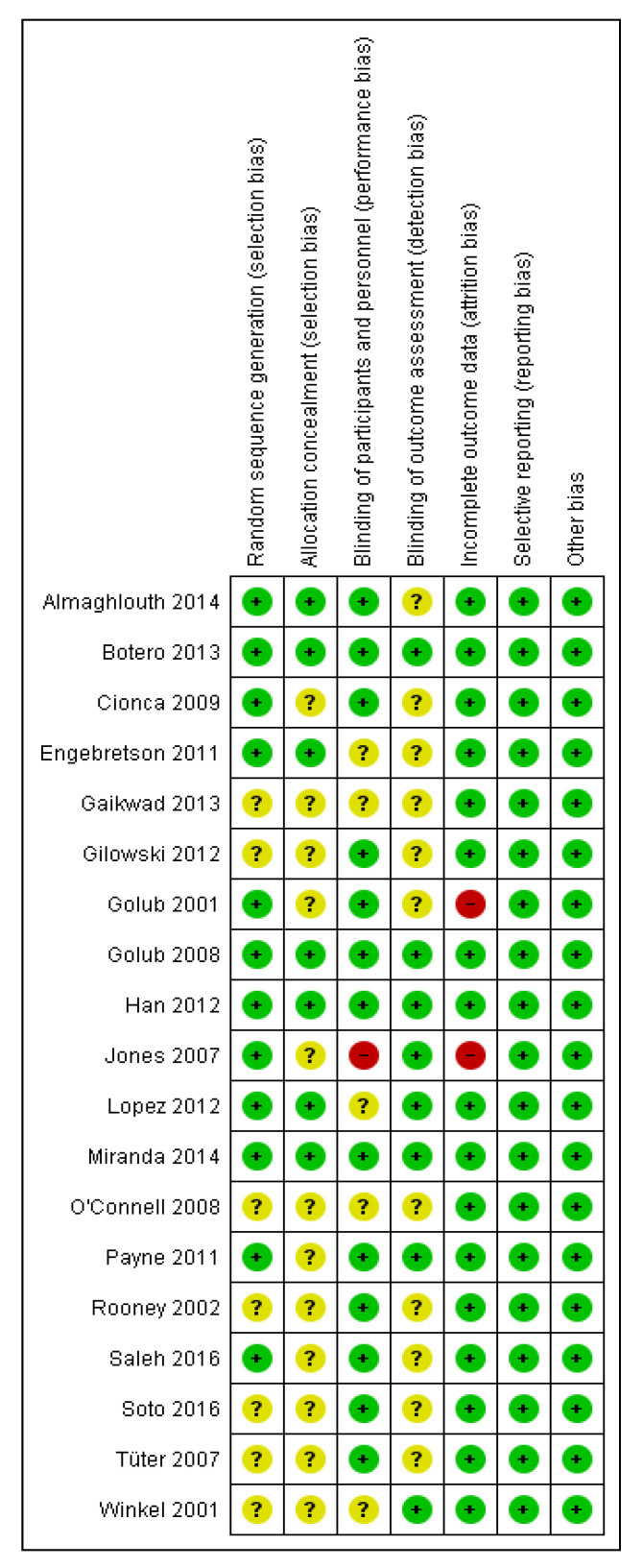
Risk of bias graph for studies assessing mechanical debridement with antibiotics in the treatment of periodontitis.

The risk of bias assessments for each included study are summarized in Figure 2. We judged 12 studies at low risk of random sequence generation (selection bias) because they reported using computer generated random numbers [19,20,21,22,25,26,27,28,29,30,31,33]. The remaining seven studies were unclear on how they generated the random allocation sequence [13,15,23,24,32,34,35].

We judged six studies at low risk of allocation concealment (selection bias) because four reported use of opaque, sealed, and coded envelopes to conceal the assignment [19,20,29,30] and two described the assignment as being concealed and allocation visually indistinguishable [22,27]. Thirteen studies were unclear on how they concealed allocation [15,21,23,24,25,26,28,31,32,33,34,35]. 

We judged 13 studies at low risk of performance bias because they reported adequate masking of participants and personnel (dentist, dental technician, and dental assistant) [13,19,20,21,24,25,26,27,30,31,32,33,35]. Information provided by the authors of five studies was unclear [15,22,23,29,34]. We judged one study at high risk of performance bias because the dentist, dental technician, dental assistant, and participants, were not masked [28]. 

We judged eight studies at low risk of detection bias because they described an adequate method of blinding outcome assessors [20,26,27,28,29,30,31,34]. The remaining 11 studies were unclear of detection bias [13,15,19,21,22,23,24,25,32,33,35].

We judged 17 studies at low risk of attrition bias since they had low attrition [13,15,19,20,21,22,23,24,26,27,29,30,31,32,33,34,35]. We judged two studies at high risk of attrition bias because they had more than 15% of participants not completing the study and did not impute missing data [25,28].

We judged all the 19 included studies at low risk of reporting bias because they reported all expected outcomes as pre-specified in their methods. 

We did not identify any other risk of bias in any of the 19 included studies.

### 3.4. Effects of Interventions

The GRADE Summary of Findings Table summarizes the results for four outcomes (Pocket depth (mm) at three months, HbA1c, MMP-8, and CRP levels) (Table 2). The detailed results for each of the primary and secondary outcomes follows. 

### 3.5. Primary Outcomes

#### 3.5.1. Cytokines/Interleukins 

Three studies reported on IL-1β [15,19,26]. One study [19] assessed 40 participants (19 receiving antibiotic and remaining 21 placebo) and observed peak values of IL-1β in four patients at baseline (values ranging from 4.8 to 226.5 pg/mL), but no peak values were detected in any of the patients after three months. Another study [26] assessed gingival crevicular fluid (GCF) IL-1β levels in 128 postmenopausal women (64 in subantimicrobial-dose doxycycline (SDD) antibiotic group and 64 in placebo group) with chronic periodontitis, however there was no significant difference in the median GCF IL-1β levels between the two treatment groups after one-year or two-years’ time-point, as reported by the study authors. The third study [15] evaluated 30 participants with type 2 diabetes and periodontitis (15 intervention and 15 placebo) but found no reduction in the concentration of IL-1β between baseline and three months (it remained at mean (standard error [SE]) of 0.3(0.3) pg/mL); however, the study authors did not give separate results for the intervention and placebo groups.

Three studies reported IL-6 [15,19,31]. One study [19] reported that a participant had a peak value of IL-6 of 216.3 pg/mL at baseline out of 40 periodontally diseased participants but there were no participants with peaked values after three months. Meanwhile, it is not clear whether the participants received antibiotic or placebo. The second study [15] reported a decrease in IL-6 in the mean (SE) of 2.1(0.3) at baseline to 1.1 (0.2) at 3 months (p = 0.005) for 30 participants with type 2 diabetes and periodontitis; however, the authors did not report separate values for the intervention and control groups except to say that there were no significant differences observed between treatment groups. The third study [31] found no significant difference in IL-6 level between the antibiotic group (SDD) and placebo group in 128 post-menopausal women with chronic periodontitis after two years (40% SDD versus 46% placebo; odds ratio [OR] 0.72, 95%CI: 0.30 to 1.69, p = 0.4).

One study [19] reported a peak value in IL-8 of 38.3 pg/mL at baseline for one participant, which rose to 39.6 after three months; but it is not clear whether the participant received antibiotic or placebo. Another study [15] reported a slight increase in IL-8 from mean (SE) of 9.0 (1.1) at baseline to 10.6 (2.5) at three months (p = 0.621) for 30 participants with type 2 diabetes and periodontitis; however, the study did not give separate values for intervention and placebo groups except to say there were no significant differences observed between treatment groups.

#### 3.5.2. C-Reactive Protein (CRP)

Five studies reported on CRP [13,19,29,31,35]. From our GRADE assessments, we found little or no antibiotic effect on CRP levels (low certainty of evidence after downgrading two levels for indirectness). In one study [19], peak values of CRP were observed in four participants at baseline (values ranging from 13.4 to 52.6 µg/mL) and in three participants at three months (14.1 to 33.4 µg/mL); however, the study authors gave no indication of how many of the participants with these peak values received antibiotics. Another study [29] assessed CRP levels in participants with metabolic syndrome where 82 participants received amoxicillin and metronidazole while 83 participants received placebo. Although the mean CRP levels decreased significantly at 9 and 12 months, there were no significant differences between the two treatment groups at three months. In a third study [31], the SDD antibiotic intervention significantly reduced median hs-CRP by 18% over a period of two years compared to the placebo and the ratio of medians of SDD versus placebo (0.82, 95%CI: 0.70 to 0.97, p = 0.02). A fourth study [13] also assessed the effect of the SDD antibiotic on high-sensitivity CRP (hs-CRP) compared to placebo; however, although there were significant improvements between baseline and six weeks in both groups, there was no significant difference between groups at six weeks (p = 0.628). The fifth study [35] reported hs-CRP at baseline and 12 weeks and found that treatment with azithromycin antibiotic decreased the hs-CRP from 4.33 mg/L to 2.99 mg/L while the placebo treatment increased from 4.59 mg/L to 5.61 mg/L. Although they did not compare these intra-group changes, they found no significant differences between groups at 12 weeks (p = 0.4630); we could not calculate the 95%CI for mean difference since standard deviations were not reported. 

#### 3.5.3. MMP-8

Four studies reported MMP-8 [13,24,26,27]. From our GRADE assessments, we found little or no impact of antibiotic therapy on MMP-8 levels (very low certainty of evidence after downgrading one level for imprecision due to low sample size and two levels for indirectness). One study [24] randomized 34 participants (17 receiving SDD and 17 receiving placebo) with chronic periodontitis and type 2 diabetes; although MMP-8 decreased significantly between baseline and three months among the SDD group while an increase was observed in the placebo group, there was no significant difference between the two groups according to the study authors. In the second study [26], the SDD significantly reduced the odds of increased MMP-8 levels by 60% compared to placebo during the two-year period of study (OR 0.40, 95%CI: 0.21 to 0.77, p = 0.006) among the 128 postmenopausal women with periodontitis (64 in each group). In the third study [27], 36 participants with generalized chronic periodontitis (18 receiving azithromycin and 18 receiving placebo) showed no significant differences in GCF MMP-8 concentration between the two treatment groups from baseline to six months. The fourth study [13] demonstrated no significant differences in MMP-8 between the SDD and placebo group among the 36 participants with chronic periodontitis at pre- and post- six-week time points, according to study authors.

#### 3.5.4. TIMP-1

One study [31] reported TIMP-1 and found no significant difference between the antibiotic group (SDD) and placebo group: TIMP-1 among the 128 postmenopausal women with chronic periodontitis after two years; ratio of medians of SDD versus placebo (0.96, 95%CI: 0.78 to 1.18, p = 0.7), according to study authors.

#### 3.5.5. HbA1c

Seven studies reported HbA1c levels [15,20,22,23,24,28,30]. From our GRADE assessments, we found little or no antibiotic effect on HbA1c levels (very low certainty of evidence after downgrading one level for high risk of bias and two levels for indirectness). One study [23] found no significant difference in HbA1c levels (%) at four months of treatment between the SDD and placebo groups among the 50 participants with type 2 diabetes and chronic generalized periodontitis (Mean (SD) 7.00 (0.76) SDD versus 7.11 (0.99) Placebo; p = 0.710); however, the different sample sizes in each treatment group at four months were not given. The second study [24] also found no significant difference in HbA1c (%) at three months after treatment with SDD compared to placebo among 34 patients with type 2 diabetes and chronic periodontitis (Median (interquartile range (IQR)) 6.3 (5.5 to 7.3) SDD versus 6.7 (6.3 to 7.7) Placebo; p = 0.8). The third study [28] evaluated the effect of SDD versus placebo after four months of treatment among 165 veterans (83 receiving SDD antibiotic and 82 placebo) with periodontitis and poorly controlled diabetes; there was no significant difference in the percent achieving either HbA1c decreases of either > 0.5 or > 1.0 (55% versus 52% (p = 0.38) or 41% versus 34% (p = 0.31), respectively). In the fourth study [20], the antibiotic group had a reduction of 0.8% versus 0.3% in the placebo group at nine months; however, no comparison p-value was calculated between the two treatment groups. In the fifth study [22], the mean HbA1c after three months decreased by 0.9% (7.2% to 6.3%) in the SDD group, increased slightly by 0.3% (7.5% to 7.8%) in the ADD group, but remained the same at 8.5% in the placebo group; there were no significant differences between the antibiotic groups and the placebo, according to the study authors. The sixth study [30] assessed metronidazole plus amoxycillin versus placebo among 58 (29 per group) participants with type 2 diabetes and periodontitis but found no significant difference in HbA1c levels (%) between the two treatment groups at baseline, three months, six months, and after one year of treatment (p = 0.35, 0.55, 0.33, and 0.62, respectively). The seventh study [15] found no significant difference in HbA1c (%) improvement between the SDD group (1.5%) and placebo group (0.9%) after three months among 30 participants with periodontitis and type 2 diabetes.

### 3.6. Secondary Outcomes

#### 3.6.1. Probing Depth (PD) 

##### (a) PD (mm)

**Figure 3:**
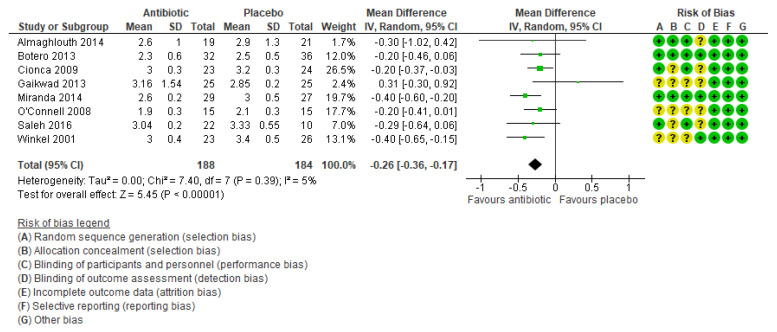
Meta-analysis of probing depth (in mm) after three months for studies assessing mechanical debridement with antibiotics in the treatment of periodontitis.

**Figure 4:**
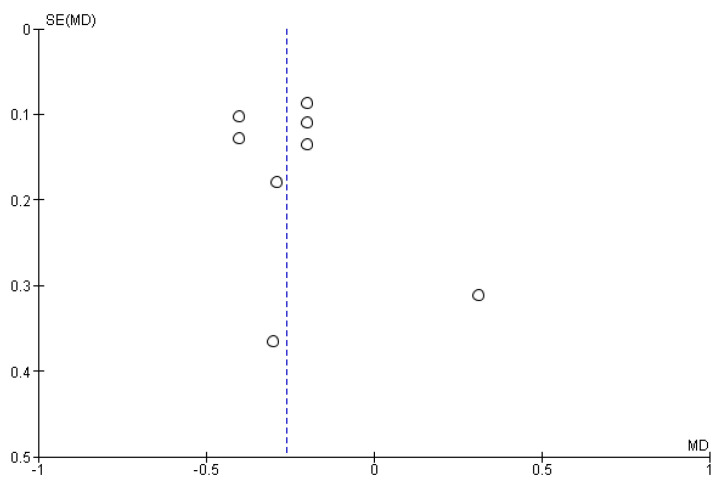
Funnel plot for the meta-analysis of probing depth (in mm) after three months of treatment with either antibiotics or placebo among adult chronic periodontitis patients.

Eight trials [15,19,20,21,23,30,33,34] measured PD (mm) at three months and a random effects meta-analysis of their results yielded a statistically significant antibiotic effect of reducing PD by 0.26 mm (mean difference [MD] −0.26, 95% confidence interval [CI]: −0.36 to −0.17, n = 372 participants, 8 trials, Figure 3) and there was no significant heterogeneity between trials (Chi^2^ = 7.40, degrees of freedom [df] = 7, p = 0.39, I^2^ = 5%). A funnel plot was constructed and it showed no evidence of publication bias (Figure 4). From our GRADE assessments, this was moderate certainty of evidence after downgrading one level for indirectness since the studies were done mainly in developed countries only. 

One trial [24] also assessed PD (mm) at three months but there were no significant differences between treatment groups; the results are only reported in a figure (box and whisker plot) from which values for analysis cannot be accurately extracted. A second trial [27] measured PD reduction (in mm) from baseline to three months and found no significant difference between antibiotic and placebo groups (MD 0.25, 95% CI -0.05 to 0.55, n = 28 participants). A third trial [13] found statistically significant improvements in PD (mm) after six weeks of treatment in favor of the antibiotic group compared to placebo (Median (IQR) of 3.45 (3.24 to 3.69) mm SDD versus 3.78 (3.52 to 4.2) mm Placebo, p = 0.034, n = 36 participants (18 per group)). A fourth trial [30] assessed the antibiotic effect at six months and one year but found no significant difference between groups (data not reported). A fifth trial [32] reported the mean percent of sites with low (0–3mm) and high (>=6mm) pocket depth in the four treatment groups at baseline, one, three, and six months. Since the study authors did not report the pocket depth (in mm), their results are not reported here.

#### 3.6.2. Clinical Attachment Level (CAL)

**Figure 5:**
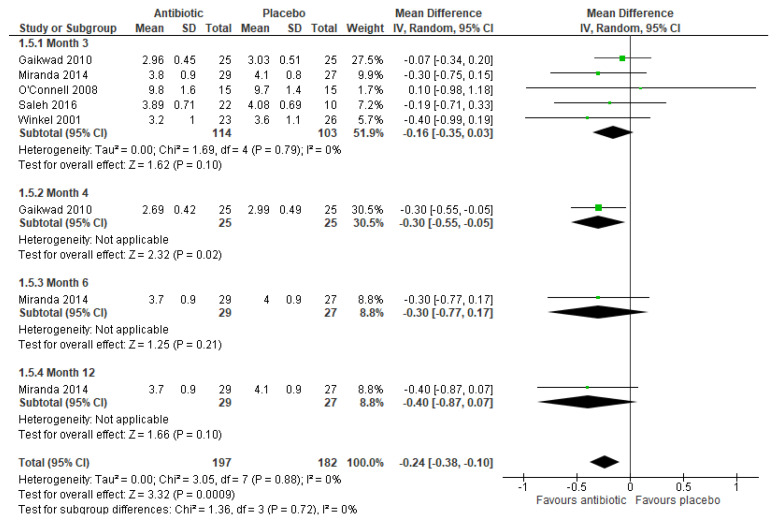
Meta-analysis of clinical attachment level (mm) after three months for studies assessing mechanical debridement with antibiotics in the treatment of periodontitis.

Nine trials reported CAL [13,15,21,23,24,27,30,33,34]. Five trials [15,23,30,33,34] measured CAL (mm) at three months and a random effects meta-analysis of their results showed no significant difference in CAL(mm) (MD −0.16, 95% CI: −0.35 to 0.03, n = 217 participants, 5 trials, Figure 5) and there was no significant heterogeneity between trials (Chi^2^ = 1.69, df = 4, p = 0.79, I^2^ = 0%). One trial [23] also measured CAL (mm) at four months and found a significant reduction in favor of the antibiotic group (MD −0.30, 95%CI: −0.55 to −0.05, 50 participants). However, another trial [30] did not find significant difference between treatment groups at both month 6 (MD −0.30, 95%CI: −0.77 to 0.17, 56 participants) and month 12 (MD −0.40, 95%CI: −0.87 to 0.07, 56 participants, Figure 5).

**Figure 6:**
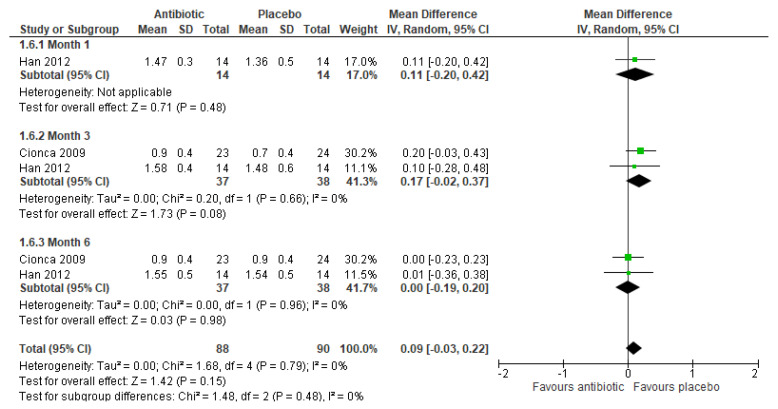
Meta-analysis of reduction in mean clinical attachment level (mm) after three months for studies assessing mechanical debridement with antibiotics in the treatment of periodontitis.

Two studies [21,27] measured the reduction in mean CAL (mm). The first study [27] found no significant differences between the treatment groups at month 1 (MD 0.11, 95%CI: −0.20 to 0.42, 28 participants). A meta-analysis of these two studies also found no significant differences between the treatment groups at month 3 (MD 0.17, 95%CI: −0.02 to 0.37, 75 participants, 2 studies) and month 6 (MD 0.00, 95%CI: −0.19 to 0.20, 75 participants, 2 studies); there was no significant heterogeneity between the two studies (I^2^ = 0%) (Figure 6).

One study [24] found no significant difference in CAL between groups (values only given in a figure) and another study [13] found no significant difference in CAL (mm) between the antibiotic and placebo groups after six weeks of treatment (Median (IQR) of 3.97 (3.75 to 4.10) mm SDD versus 4.0 (3.66 to 4.46) mm Placebo, p = 0.521, n = 36 participants (18 per group)). 

## 4. Discussion

We assessed the effectiveness of systemic antibiotics as an adjunctive therapy to mechanical debridement in the changes of inflammatory systemic biomarkers as well as clinical parameters among adult patients with chronic periodontitis. We included 18 randomized controlled trials consisting of 1350 adult participants in this systematic review and we found very little impact of antibiotics on changes in blood levels of inflammatory biomarkers such as MMPs, TiMPs, Cytokines/Interleukins (IL-1β, IL-6, and IL-8), CRP, HbA1c, and clinical attachment level. However, a meta-analysis of eight studies demonstrated a mean reduction of 0.26 mm in the periodontal pockets at three months (moderate certainty of evidence) in favor of the antibiotics. In meta-analyses, we did not detect substantial heterogeneity between studies and the studies generally had either unclear or low risk of bias. The majority of the studies were conducted in developed countries, mainly in Europe and the USA.

To our knowledge, this is the first systematic review to determine the effects of adjunctive antibiotics on systemic biomarkers. Several systematic reviews have assessed the effects of adjunctive antibiotics among patients with periodontitis; however, they assessed clinical outcomes only. From the two most recently published systematic reviews [36,37], the first [36] assessed the effect of adjunctive systemic antimicrobials on clinical outcomes of periodontitis in randomized controlled trials with follow-up of at least six months; they found that systemic antibiotic therapy with metronidazole and amoxycillin significantly improved probing pocket depth as well as clinical attachment level. The other systematic review [37] assessed adjunctive antibiotics in patients with untreated chronic periodontitis and also found significant effects of systemic antibiotics on pocket depth reduction at 3, 6, and 12 months, as well as on clinical attachment level gain after three months of treatment. This is in agreement with our findings of a statistically significant reduction in pocket depth at three months; however, our results did not find significant effects in clinical attachment level. The difference in our results is probably because we excluded studies before the year 2000 and did not separate analyses for different types of antibiotics as done in the other two systematic reviews.

In this systematic review, we included adult participants aged 18 to 75 years with moderate to advanced chronic periodontitis with or without comorbidities mainly from developed countries. Only one study was from India and none were from the African continent. This therefore limits the generalizability of our findings to young people and other population groups and regions not included in this review. Numerous included studies did not report results in a clear, tabular form with separate results for intervention and control groups [13,19,20,22,24,27,29,31]. In our GRADE assessment, we downgraded the certainty of evidence due to the lack of generalizability and the poor reporting of results. Overall, there is insufficient evidence to make general conclusions on the effects of adjunctive antibiotics on systemic biomarkers in the treatment of periodontitis. The use of antibiotics as an adjunct to mechanical debridement is seen to be satisfactory even though their results have been deemed as having little difference between the antibiotic and placebo groups in the outcomes concerning pocket depth reduction (at three months), the reduction of HbA1c, MMP-8, and CRP. The fact that antibiotic treatment disrupts the inflammatory pathways of chronic periodontitis serves as a justification for the adjunctive use of antibiotics with scaling and root planing (SRP) even if the clinical benefit is minor. The adjunctive use of 400 mg or 250 mg of metronidazole plus 500 mg of amoxicillin for 14 days provided clinically relevant benefits over SRP exclusively in the treatment of generalized chronic periodontitis [38]. Antibiotics are not a panacea for all non-responsive situation; however, adjunctive antibiotic use together with mechanical debridement provides effective control in chronic periodontitis [39].

We minimized bias in the review process in a number of ways. We conducted a comprehensive literature search of all relevant electronic database and consulted experts to identify grey or unpublished literature and relevant information. Our search was not language-restricted and in fact, we included one French study for which we received professional translation [35]. We also screened reference lists of included articles to identify potentially eligible studies. At least two review authors independently scrutinized and selected articles for inclusion in the review using pre-specified eligibility criteria. Articles were assessed for risk of bias and data were extracted in duplicates. However, there were some potential biases in the process of this systematic review. For instance, we did not perform separate analyses of studies with and without comorbidities and we are therefore unable to determine if there are differences in effects of systemic antibiotics between individuals with and without comorbidities. There is a difference in the host response to antibiotics in a systemically healthy individual compared to an individual with a co-morbidity during periodontal treatment [40]. We also did not compare the effects of different antibiotics and different dosages on the outcomes. Therefore, the results from this review will need cautious interpretation because of the broad grouping of participants and interventions. Future updates of this systematic review will need to determine if such differences exist.

## 5. Conclusions

### 5.1. Implications for Practice

The limited available evidence shows that adjunctive administration of systemic antibiotics for a minimum of three months may improve pocket depth clinical parameters compared to mechanical debridement alone, among adults with chronic periodontitis. Similarly, the extended two-year duration of adjunctive SDD with mechanical debridement may improve systemic CRP and MMPs serum levels.

### 5.2. Implications for Research

The included trials reported on the following outcomes: Serum/blood levels of MMPs, TIMPs, Cytokines, CRP, IL-1β, IL-6, IL-8, CRP, HbA1c, PD, and CAL. Further rigorous large randomized controlled trials of high quality would be beneficial to assess the effect of adjunctive antibiotics administration on systemic biomarkers in chronic periodontitis. Most of the included trials are of poor methodological quality and the results were portrayed in graphic displays, making it difficult to extract the data accurately. This limits the applicability of the result in clinical evidence-based practice.

## Figures and Tables

**Table 1:** Characteristics of included studies on the effect of antibiotics in addition to mechanical debridement among adults with chronic periodontitis.

Study ID	Country and Setting	Patients	Trial Duration	Intervention	Control
Almaghlouth 2014 [19]	School of Dental Medicine of the University of Geneva, Switzerland	40 patients with moderate-to-advanced periodontitis, aged 25–70 years	3 months	500 mg Metronidazole+ 375 mg Amoxicillin three times a day for 7 days	No placebo
Botero 2013 [20]	San Vicente de Paul Hospital Medellin, Colombia, South America	105 adults diagnosed with moderate periodontitis and diabetes	18 months	Azithromycin tablet 500 mg daily for 3 days (Group 1: Az+Scaling, Group 2: Az+Prophylaxis)	Placebo
Cionca 2009 [21]	School of Dental Medicine, University of Geneva, Switzerland	51 adult patients with moderate to advanced periodontitis, between ages of 25–70 years	1 year	500 mg Metronidazole & 375 mg Amoxicillin t.i.d × 7 days	Placebo capsules of 500 mg and 375 mg t.i.d. × 7 days
Engebretson 2011 [22]	Naomi Berrie Centre and Department of Periodontics, Columbia University Medical Centre, USA	45 Type 2 diabetes patients with chronic periodontitis, aged 50–56 years.	3 months	Group 1: 20 mg Sub-antimicrobial dose doxycycline (SDD) b.i.d × 3 months Group 2: 100 mg ADD daily × 3 months	Placebo daily × 3 months
Gaikwad 2013 [23]	Department of Periodontics, Tatyasaheb Kore Dental College, India	50 diabetes patients with chronic generalised periodontitis aged 30–70 years.	3 months	100 mg Doxycycline once daily for 15 days	No placebo
Gilowski 2012 [24]	Medical University of Silesia, Katowice, Poland	34 type 2 diabetes patients with chronic periodontitis, aged 36–68 years	3 months	20 mg doxycycline hydrochloride three times a day for 3 months	No placebo
Golub 2008 [26] and Payne 2011 [31]	University of Nebraska Medical Centre College of Dentistry and the School of Dental Medicine at Stonybrook University, Stoney Brook, USA	128 Osteopenic post-menopausal women with moderate to advanced periodontitis between the ages of 45–70 years	2 years	20 mg Sub-antimicrobial dose doxycycline (SDD) × 3 times a day for 2 years	20 mg Placebo × 3 times a day for 2 years
Golub 2001 [25]	Department of Oral Biology & Pathology, School of Dental Medicine, State University of New York at Stony Brook, USA	174 adult patients with chronic periodontitis aged 18–75 years	9 months	Group 1–4 Doxycycline 20 mg × 12 weeks (in different combinations)	Placebo × 12 weeks
Han 2012 [27]	Department of Periodontology, School of Dentistry, Ege University, Izmir, Turkey	36 patients with severe generalised chronic periodontitis in the age range of 35–54 years	6 months	500 mg Azithromycin once daily × 3 days	500 mg Placebo once daily for 3 days
Jones 2007 [28]	All four departments of Veterans Administration facilities at Greater Boston, USA	165 diabetes patients with periodontitis, mean (SD) age of 59.1(11) years	4 months	100 mg doxycycline daily for 14 days	Usual care with no placebo
Lopez 2012 [29]	Dr. Eloisa Diaz Dental Center, San José Hospital, Santiago, Chile	165 patients with Metabolic Syndrome (MetS) having periodontitis, between the ages of 35–65 years	1 year	250 mg Metronidazole t.i.d AND 500 mg Amoxicillin t.i.d for 7 days	250 mg placebo t.i.d AND 500 mg placebo t.i.d for 7 days
Miranda 2014 [30]	Department of Periodontology, Dental Research Division, Guarulhos University, Sao Paulo, Brazil	58 Type 2 diabetes patients with generalized chronic periodontitis, aged 35 years or more.	1 year	400 mg Metronidazole+ 500 mg Amoxycillin three times a day for 14 days	Placebo
O’Connell 2008 [15]	Department of Oral Surgery & Periodontology, University of São Paulo Ribeiro-Preto, Brazil	30 Type 2 diabetes patients with periodontitis, aged 46–70 years	3 months	100 mg Doxycycline once daily for 2 weeks after an initial dose of 200 mg	Placebo once daily for 2 weeks after initial dose of placebo
Rooney 2002 [32]	Department of Periodontology at Bristol Dental School and Hospital, USA	66 patients with advanced chronic periodontal disease between the ages of 20–45 years	6 months	Group 1 (AM): 250 mg Amoxycillin (capsule) and 200 mg metronidazole (tablet). Group 2 (PM): 200 mg metronidazole (tablet) and placebo (lactose capsules). Group 3 (AP): 250 mg Amoxycillin (capsule) and placebo (calcium lactate tablets).	Placebo (lactose capsules) and placebo (calcium lactate tablets).
Saleh 2016 [33]	Oral Health Centre, University of Western Australia, Australia	37 adults with generalized moderate to advanced chronic periodontitis, age of 30 years and older	3 months	Group 1: 500 mg Amoxicillin and 200 mg Metronidazole administered every 8 h for 7 days Group 2: 500 mg Azithromycin administered every 8 h for 7 days	Placebo capsules were administered every 8 h for 7 days
Soto 2016 [35]	Two university clinics in the city of Cali (Colombia), and the Universidad del Valle (university of Valle)	81 patients with moderate to severe chronic periodontitis, aged between 25 and 70 years	12 weeks	500 mg Azithromycin per day, for 5 days	Placebo
Tuter 2007 [13]	Department of periodontology of Gazi University, Ankara, Turkey	36 patients with both chronic periodontitis and coronary artery disease (CAD), and age < 70 years	6 weeks	20 mg Sub-antimicrobial dose doxycycline (SDD) three times daily for 6 weeks	20 mg Placebo, three times daily for 6 weeks
Winkel 2001 [34]	Clinic for Periodontology Amsterdam and the Clinic for Periodontology Utrecht, The Netherlands	49 patients with generalised severe periodontitis, mean age of at least 40 years	6 months	375 mg Amoxicillin in combination with 250 mg metronidazole to be taken every 8 h for next 7 days was given.	Similar placebos every 8 h for next 7 days was given

**Table 2:** GRADE Summary of Findings Table for studies assessing mechanical debridement with antibiotics in the treatment of periodontitis.

**Summary of Findings Table:**						
**Antibiotic compared to placebo in the treatment of chronic periodontitis**						
**Patient or population**: Adult patients diagnosed with chronic periodontitis **Setting**: Academic dentistry hospitals or clinics in developed countries **Intervention and Comparison**: Antibiotic compared placebo or no antibiotic						
**Outcomes**	Anticipated Absolute Effects * (95% CI)		**Relative Effect** **(95% CI)**	**№ of Participants** **(studies)**	**Certainty of the Evidence** **(GRADE)**	**Comments**
	**Risk with Placebo**	**Risk with Antibiotic**				
Pocket depth (mm) at 3 months	The mean pocket depth (mm) at 3 months was **0**	MD **0.26 lower** (0.36 lower to 0.17 lower)	-	372 (8 RCTs)	⨁⨁⨁◯ MODERATE ^a^	Meta-analysis of eight studies with 372 participants show an antibiotic effect of reducing pocket depth by 0.26 mm compared to the placebo (moderate certainty of evidence). One other study found reduction in favour of the antibiotics, however results were presented as medians and ranges. Three other studies found little or no difference between the antibiotic and placebo groups.
Glycosylated Haemoglobin (HbA1c)	See comment	not pooled	-	487 (7 RCTs)	⨁◯◯◯ VERY LOW ^b,c^	Six studies with 382 participants found little or no difference in the effect of antibiotics on haemoglobin levels compared to the placebo group. One study with 105 participants was unclear on the differences between the antibiotic and placebo groups.
MMP-8	See comment	not pooled	-	234 (4 RCTs)	⨁◯◯◯ VERY LOW ^c,d^	Three small studies with 106 participants found little or no impact of antibiotic therapy on MMP-8 levels. One study with 128 participants found a 60% reduction in favour of antibiotics during a 2-year period.
C-Reactive Protein (CRP)	See comment	not pooled	-	504 (5 RCTs)	⨁⨁◯◯ LOW ^c^	Three studies with 282 participants found little or no difference in CRP levels between the antibiotic and placebo groups. One study with 40 participants was unclear on the inter-group differences. One study with 182 participants found a small antibiotic effect of 18% decrease.
* The risk in the intervention group (and its 95% confidence interval) is based on the assumed risk in the comparison group and the **relative effect** of the intervention (and its 95% CI). **CI**: Confidence interval; **MD**: Mean difference						
**GRADE Working Group grades of evidence** **High certainty**: We are very confident that the true effect lies close to that of the estimate of the effect **Moderate certainty**: We are moderately confident in the effect estimate: The true effect is likely to be close to the estimate of the effect, but there is a possibility that it is substantially different **Low certainty**: Our confidence in the effect estimate is limited: The true effect may be substantially different from the estimate of the effect **Very low certainty**: We have very little confidence in the effect estimate: The true effect is likely to be substantially different from the estimate of effect						

a. Downgraded for indirectness as most studies were done in developed countries and results may not be generalizable globally; b. One study (Jones 2007) had high risk of performance and attrition bias; c. Indirectness: Outcomes were reported in different forms by studies and could not be pooled in a meta-analysis. In addition most studies were done in developed countries and results may not be generalizable globally. d. Three studies had small sample sizes of below 40.

## Abbreviations

ADD: Anti-microbial dose doxycycline; CAL: Clinical attachment level; CI: Confidence interval; CRP: C-reactive protein; CAD: Coronary artery disease; FMPS: Full mouth plaque score; GCF: Gingival crevicular fluid; GI: Gingival index; HbA1c: Glycosylated hemoglobin; GRADE: Grading of recommendations assessment, development, and evaluation; IL-1β: Interleukin-1-beta; IL-6: Interleukin-6; IL-8: Interleukin-8; IQR: Inter-quartile range; MD: Mean difference; MetS: Metabolic syndrome; MMP-8: Matrix metallo-proteinase-8; OR: Odds ratio; PD: Probing depth; PPD: Probing pocket depth; PI: Plaque index; RCT: Randomized controlled trial; SD: Standard deviation; SDD: Sub-antimicrobial dose doxycycline; SCP: Severe chronic periodontitis; SE: Standard error; SRP: scaling and root-planing; TIMP-1: tissue inhibitor metallo-proteinase-1; TNF-α: Tumor necrosis factor – alpha; USA: United States of America; WHO: World Health Organization.
